# Navigating Online and in-Person Support: Views and Experiences From Survivors of Intimate Partner Violence and Abuse

**DOI:** 10.1177/10778012241270223

**Published:** 2024-08-08

**Authors:** Nicole van Gelder, Jeyna Sow, Ditte van Haalen, Iris Schoorlemmer, Margreet Knol, Eva Bouwer, Sabine Oertelt-Prigione

**Affiliations:** 1 Gender Unit, Department of Primary and Transmural Care, Radboud University Medical Center, Nijmegen, The Netherlands; 2 Kadera Aanpak Huiselijk Geweld, Zwolle, The Netherlands; 3 Department of Sex- and Gender-Sensitive Medicine, Medical Faculty OWL, University of Bielefeld, Bielefeld, Germany

**Keywords:** intimate partner violence and abuse, domestic violence and abuse, help-seeking, in-person, online

## Abstract

Various types of in-person and online support are available to women intimate partner violence and abuse (IPVA) survivors. However, we know little about the interplay between them. We investigated the transitions and interactions between these types of help and how their use can be optimized, using a mixed-methods approach (survey *N* = 107; interviews *N* = 18). Significant but weak correlations were found for specific IPVA and support types. No significant correlations were found between online and in-person help types. Almost 60% of survey participants expressed interest in blended care. Integration and optimization of online and blended care options can increase outreach and provide an enhanced, tailored help-seeking and recovery journey.

## Introduction

Intimate partner violence and abuse (IPVA) consists of psychological, physical, sexual, and economic abuse by an intimate (ex-)partner (Ali et al., 2016; Johnson et al., 2022; Van Eijkern et al., 2018; World Health Organization, 2021). Although both men and women experience IPVA, women are most frequently victimized (Van Eijkern et al., 2018). Globally, approximately 25% of women are subjected to physical and/or sexual violence by an intimate (ex-)partner in their lifetime (World Health Organization, 2021). In the Netherlands, approximately 4% of women experienced physical or sexual violence committed by their (ex-)partner in the previous 5 years (Ten Boom & Wittebrood, 2019). Exposure to IPVA has significant negative effects on various life domains, for example, on (mental) health with injuries, chronic pain, depression, and posttraumatic stress disorder (Black, 2011; Ellsberg et al., 2008).

Women seeking help from formal services, such as their general practitioner (GP) or domestic violence and abuse (DVA) organizations, often face barriers such as fear, shame, access challenges, consequences of disclosure, and system failures (O’Doherty et al., 2016; Robinson et al., 2020). Only a minority of Dutch women reports incidents to the police (15–17%), seeks medical help (23%) or help from a formal service or community agency (4–6%; Römkens et al., 2016; Ten Boom & Wittebrood, 2019).

Another option is online support, which is increasingly available in the Netherlands since the COVID-19 pandemic (Brink et al., 2021; Van Bemmel et al., 2020; Van Gelder et al., 2021b) and includes chats on websites of DVA organizations such as *Veilig Thuis* (VT; translation: Safe Home; national DVA organization) and *Fier*, the SAFE platform (Van Gelder et al., 2020) and online modules. Internationally, a similar trend can be observed with an increasing number of digital support options (Brink et al., 2021; Caridade et al., 2021; Emezue, 2020). While scientific evaluations of online interventions for IPVA survivors are relatively novel and limited, the currently available results are promising. They can reduce barriers to help-seeking due to easy accessibility, potentially available 24/7 and anonymity (Ford-Gilboe et al., 2020; Glass et al., 2021; Hegarty et al., 2019).

The survivors’ needs and obstacles in seeking and using online and in-person (offline) help have been identified (e.g., Emezue, 2020; Evans & Feder, 2016; Glass et al., 2021; Lelaurain et al., 2017; Van Gelder et al., 2021a). Online interventions such as I-DECIDE (Hegarty et al., 2019) and myPlan (Glass et al., 2021) do not aim to replace in-person help and, in addition to providing online support, often refer survivors to these support services. However, we have little knowledge about how survivors navigate these types of help. Furthermore, the help-seeking process is not linear and is influenced by various aspects, for example, the type and severity of the IPV and various intersectional personal and social factors (e.g., migration background or gender-stereotypical norms in society; Lelaurain et al., 2017). Professionals expressed interest in blended care (online and in-person support combined) and pointed to the added value of online help, since it can easily provide information and help options, while potentially representing a bridge toward in-person help (Caridade et al., 2021; Garnweidner-Holme et al., 2020; Van Gelder et al., 2021b). A stepping stone in seeking professional help is important (Evans & Feder, 2016) and an online platform can be the first place for disclosure and referral (Van Bemmel et al., 2020).

Since both in-person and online care are important in supporting IPVA survivors, the current study investigates the interplay between these types of help and the mechanisms behind it. We focus explicitly on the direction from online to in-person support, as online support is often aimed at lowering the threshold for in-person support, but also take other directions into account, as we know that help-seeking is a complex process that can encompass seeking various types of help over time. We further explore optimizing the use of both forms of help, with respect to the different functions that they may have in help-seeking journeys.

## Method

### Study Design and Data Acquisition

In this exploratory mixed-methods study, we combined questionnaire and interview data to investigate the research questions:

- What steps do women who have experienced IPVA take in the transition from online to in-person help and how do they experience these steps?
- Does the use of online help lead women to use in-person help?
- How can the use of online and in-person help be optimized?

Study participants were divided into three groups: survivors who only had experience with online support (ONG), survivors who only had experience with in-person support (IPG), and survivors who had experience with both online and in-person help: the hybrid group (HG).

The entire study was conducted in Dutch, and all participants received a digital information letter and provided digital consent. The quantitative data were collected via an online survey. The survey gathered information about gender identity, age, and educational level, and contained multiple choice questions, Likert scales, and open questions about the participants’ experiences with online and in-person help. For example, “What do/did you need most when using online support?” (Supplemental File A.1).

The qualitative part consisted of semistructured online interviews and open survey questions. The interviews lasted 30–45 min and audio recordings were typed out *verbatim.* Two researchers with backgrounds in criminology and psychology (DvH; JS) conducted the interviews using a flexible interview guide with questions such as: “Are there things that you miss(ed) in online help that are present in in-person help and vice versa?” (Supplemental File A.2). We achieved code and meaning saturation with 18 interviews (Hennink et al., 2017).

### Ethical Approval

The Committee for medical-ethical testing (CMO region Arnhem–Nijmegen) provided a declaration stating this study does not require formal approval from them or another recognized medical-ethical review committee (dossier 2021-13226).

### Recruitment and Study Population

For both the survey and interviews, participants were mainly recruited online: through open posting on the social media accounts (Instagram, Twitter, and Facebook) of SAFE (online intervention for women IPVA survivors) and Kadera (DVA organization), and through LinkedIn. Kadera also recruited participants among their clients who received various types of in-person help. At the end of the online survey, we asked participants if we could also approach them for an interview. However, we also disseminated calls to register for an interview in our online recruitment strategy, meaning that someone could also participate in an interview without having filled in the survey.

Inclusion criteria were: identifying as a woman and IPVA survivor, aged 18+ years, proficient level of Dutch, and having experience with online and/or in-person support. The survey was online in the same period that we conducted interviews, all data were gathered between 1 December 2021 and 28 February 2022.

### Analysis

Quantitative data were analyzed using SPSS version 25 (IBM Corp., 2017). We used descriptive analysis and group comparisons. Pearson's correlation was used to assess interactions between demographics, type of IPVA and types of help.

Qualitative survey data were summarized and interpreted through qualitative content analysis (Henning et al., 2004). The interview data were coded independently by two researchers (JS and NvG) using open thematic coding with an explanation comment for every code (Ayres, 2014) in Atlas.ti version 9.1.6 (Friese, 2011) and interpreted through qualitative content analysis (Henning et al., 2004). To ensure no relevant data were omitted a third researcher (IS) coded the first nine interviews as well. The final codebook was established through six discussion rounds between the coders. Furthermore, in interpreting the content of the interviews, JS and NvG discussed the data with two professionals from Kadera (MK and EB) to include the DVA professionals’ perspectives. To check for the completeness of the data, all interviews were reread using the final codebook. Intercoder reliability was checked, resulting in 82% intercoder agreement.

To integrate the survey and interview data, we compared the outcomes of the survey to the outcomes of the interviews. The survey outcomes form the base of the results while the interview outcomes add in-depth insights. Therefore, if applicable, we described the survey and interview outcomes together per theme.

## Results

In total, 107 women completed the survey and 18 women took part in the interviews. Fourteen women completed both the survey and interview, thus we included 111 unique participants in this study. All participants identified their sex as “female” and their gender as “woman,” except for one participant who answered “male” for sex and “woman” for gender, and one participant who answered “female” for sex and “I’d rather not say” for gender. The mean age for the survey group and interview group was 43 years. Psychological abuse was the most reported type of IPVA for the survey (98.1%; Table 1) and interview group (100%; Table 2). We identified four groups corresponding to types of help experience: HG (online and in-person; survey *N* = 42; interview *N* = 16), in-person only group (IPG; survey *N* = 53; interview *N* = 2), online only group (ONG; survey *N* = 4; interview *N* = 0), and no-help group (survey *N* = 8; interview *N* = 0). While the inclusion criteria demanded participants to have experience with at least one type of support, there were eight survey participants who stated that they did not receive any help. Even though we sought survivors’ experiences with various types of support, we also know that many survivors do not reach out to professional help (at an early stage) and therefore, we thought it was important to include these eight participants after all to receive more information on the obstacles they face in help-seeking.

**Table 1.** Survey Participants’ (*N* = 107) Demographics.

Age	Educational level	Province	IPVA type	Type of support
Mean age = 43	Secondary education: *N* = 4	Drenthe: *N* = 2	Psychological: *N* = 105	Offline only: *N* = 53
Range = 21–74	Vocational education: *N* = 37	Flevoland: *N* = 5	Physical: *N* = 82	Online only: *N* = 4
Age category	Higher vocational education: *N* = 49	Friesland: *N* = 1	Economical: *N* = 61	Hybrid (online and offline): *N* = 42
21–29: *N* = 11	University: *N* = 16	Gelderland: *N* = 14	Sexual: *N* = 44	No help: *N* = 8
30–39: *N* = 27	Postdoctoral: *N* = 1	Groningen: *N* = 5		
40–49: *N* = 40		Limburg: *N* = 3		
50–59: *N* = 25		Noord-Brabant: *N* = 11		
60–69: *N* = 3		Noord-Holland: *N* = 14		
70–74: *N* = 1		Overijssel: *N* = 16		
		Utrecht: *N* = 6		
		Zeeland: *N* = 5		
		Zuid-Holland: *N* = 25		

**Table 2.** Interview Participants’ (*N* = 18) Demographics.

Age	Educational level	Province	IPVA type	Type of support
Mean age: 43	Secondary education: *N* = 1	Drenthe: *N* = 0	Psychological: *N* = 18	Offline only: *N* = 2
Range: 26–75	Vocational education: *N* = 6	Flevoland: *N* = 1	Physical: *N* = 16	Online only: *N* = 0
Age category	Higher vocational education: *N* = 11	Friesland: *N* = 0	Economical: *N* = 10	Hybrid (online and offline): *N* = 16
26–29: *N* = 2	University: *N* = 0	Gelderland: *N* = 4	Sexual: *N* = 11	No help: *N* = 0
30–39: *N* = 5	Postdoctoral: *N* = 0	Groningen: *N* = 1		
40–49: *N* = 7		Limburg: *N* = 0		
50–59: *N* = 3		Noord-Brabant: *N* = 0		
60–69: *N* = 0		Noord-Holland: *N* = 3		
70–75: *N* = 1		Overijssel: *N* = 5		
		Utrecht: *N* = 0		
		Zeeland: *N* = 0		
		Zuid-Holland: *N* = 4		

### Mapping Online and In-Person Help

#### Obstacles and Needs

Shame emerged as a major obstacle in help-seeking in general, for both survey and interview participants. The no help group (*N* = 8) reported shame as a main obstacle. This was also the case for women who eventually did receive support. A percentage of 61.2% of the women who stated they had experienced obstacles in in-person help-seeking (*N* = 85) reported shame as an obstacle. For women experiencing obstacles in online help-seeking (*N* = 82), shame was also the most reported obstacle (42.7%). The obstacle of shame was especially applicable to survey participants who reported that seeking online (*N* = 11 of 25) or in-person (*N* = 36 of 62) help was moderately to very difficult. Other prominent obstacles were feelings of guilt (survey group; online: 35.4%; in-person: 38.8%) and thinking their situation was not serious or severe enough (survey group; online: 35.4%; in-person: 31.8%). These obstacles were present in the responses of the interviewees as well when looking at help-seeking obstacles in general. Furthermore, they mentioned additional obstacles, such as fear of their (ex-)partner finding out and possible victim-blaming or disbelief.

Yeah, I think it's shame and also ‘is it really that bad?’. Maybe you’re also suppressing things and a fear of ‘what are they going to do?’. Are they going to do things that I don’t want, is it going to increase stress? … Judging maybe. (p. 120)

Specifically for online help, survey participants mentioned the obstacle of safety concerns (39%). Many interviewees said it lacks personal, “real” contact and they thought it is harder to share their feelings. This opinion was also present in the survey group: 23.2% reported online support being impersonal as an obstacle. For in-person help, survey participants mentioned practical obstacles, such as a lack of time or suitable help nearby (28.2%). This was also reflected in interview responses and interviewees also mentioned fear of losing their children (Supplemental File A.3).

With regard to needs, the survey showed that acknowledgement was an important factor in general. A percentage of 54.3% of survey participants with online experience (HG and ONG *N* = 46) and 54.7% of respondents with in-person experience (HG and IPG *N* = 95) reported this as one of their most important needs. Acknowledgment was the most discussed general need in the interviews. Other major general needs that emerged from the survey and interviews were: practical information and support, continuity of help and guidance in navigating multiple types of help, and feeling supported. Specifically for in-person help, receiving immediate (acute) care or help was a main need (survey group: 57.9%). This was also true for the interviewees and they said social interaction was important. A prominent need for online help was receiving specific tips (survey group: 69.6%). The interviews reflected this need and they also addressed the need for “real” interaction as a prominent need (Supplemental File A.3).

Maybe that it's [online help] more personalized. That you really feel like you talk to someone instead of to some automated system. For example, that you see their first name in the corner or a picture that gives an idea of the person you’re speaking with. I think that would help lower the barrier. (p. 101)

#### Advantages and Disadvantages

The advantages and disadvantages of both types of help were partially similar to the obstacles and needs (Supplemental File A.3). The interview group elaborated on this theme. Specifically for online help, interviewees mentioned being able to reread something and promoting awareness through sharing examples of IPVA. For in-person help, emotional and practical support that can be tailored to individual needs emerged as an advantage. With regard to disadvantages, a few interviewees felt that online modules did not meet their needs in communicating about their situation or their preferences in receiving information. For in-person help, some women mentioned that the amount of involved professionals and help options can be overwhelming.

I noticed it [online module with video] was too slow for me. … But it's very personal. They [professionals] said that they offer it slow paced because there are also women who do not speak Dutch that well and they need to be able to follow it as well. I’m highly educated and I really like to read, I’d have preferred to read a text. (p. 114)

There was no overarching organization that can help you with every aspect. So you have a question, you need to go left. You have a slightly different question, you need to go right. It would be good if there's something overarching that can help victims because it's very difficult. (p. 105)

#### Usage

Certain help types and IPVA types seemed to be linked. For ONG and HG,^1^ women who experienced physical abuse appeared somewhat less likely to use an internet search engine for help-seeking (*r *= .39; *p *= .008; *N* *= *46). Furthermore, survivors of economic abuse seemed slightly more likely to use the online chat of a DVA organization (*r *= .31; *p *= .036; *N* *= *46). For IPG and HG,^2^ participants who endured physical violence appeared a bit more likely to seek help from the police (*r *= .32; *p *= .002; *N* *= *95) and we found similar positive correlations between sexual IPVA and a DVA organization (in-person support) (*r *= .21; *p *= .040; *N* *= *95), and psychological IPVA and in-person help from a psychologist (*r *= .22; *p *= .031; *N* *= *95).

Also, younger women (age 21–39) were more likely to use online help than older women (age 40+). However, some women sought help before online options were (sufficiently) available. Indeed, a few women in the interview group mentioned this and discussed age as a possible obstacle to online help. In-person help was used the most for the entire study sample (Supplemental File A.3).

Maybe it's easier for younger people, they’re more used to online activities. But there are also a lot of older people who, years later, decide to leave the violent situation. And often they’re digital illiterates. They’re not familiar with it. (p. 113)

### The Dynamics Between Online and In-Person Help

#### Separate Help Systems Versus One Integrated System

We found correlations within the type of help but no significant correlations were found between online and in-person help in the HG (*N* = 42; Table 3). For online help, women who used the VT chat seemed significantly more likely to also use a DVA organization's chat (*p *= .01). Participants who used these chat options appeared significantly less likely to also use an internet search engine (*p *= .05). For in-person help, if VT was involved it was significantly more likely that a DVA organization, social work (*p *= .05) and/or police (*p *= .01) were involved as well. Furthermore, social work showed significant positive links with the GP, police and/or psychologist (*p *= .05). Computations for online (*N* = 46) and in-person forms of help (*N* = 95) separately confirmed the aforementioned results.

**Table 3.** Help Option Correlations in the Hybrid Help User Group (*N* = 42).

	1.	2.	3.	4.	5.	6.	7.	8.	9.	10.	11.
1. Internet search engine (online)	–										
2. Chat VT (online)	−0.372*										
3. Chat DVA organization (online)	−0.312*	0.433**									
4. SAFE (online)	0.021	0.226	0.000								
5. Courses or modules (online)	−0.046	−0.086	0.149	0.271							
6. VT (offline)	−0.272	0.069	0.100	0.175	−0.006						
7. DVA organization (offline)	0.059	−0.040	0.251	0.200	0.014	0.367*					
8. GP (family doctor) or GP office's mental health worker (Dutch: *POH-GGZ*; offline)	0.225	−0.240	−0.079	−0.022	0.276	0.048	−0.108				
9. Police (offline)	−0.091	0.034	−0.117	−0.043	−0.098	0.528**	0.139	0.106			
10. Social work/social team (Dutch: *buurtteam/social team*)/center for children and families (Dutch: *Centrum voor Jeugd en Gezin*; offline)	−0.100	−0.034	−0.019	0.171	0.220	0.355*	0.085	0.379*	0.336*		
11. Psychologist (offline)	−0.165	0.171	0.198	0.108	0.029	0.147	0.005	0.209	0.203	0.379*	
12. Sexual assault center (Dutch: *Centrum Seksueel Geweld*; offline)	0.142	−0.110	−0.064	−0.070	−0.076	0.199	0.279	0.129	0.164	−0.164	0.129

*Note.* Interactions between offline and online help types were analyzed in one model with Pearson's correlations. Only HG (*N* = 42) was included as all participants in this group used both online and offline help.

*Correlation is significant at the 0.05 level (two-tailed).

**Correlation is significant at the 0.01 level (two-tailed).

While online and in-person help did not show significant linkages, a part of the survey group did express certain needs and curiosity for combining these types of support. 78.3% of participants who used online help (*N* = 46) also received in-person help, mainly from their GP or GP office's mental health worker (36.1%). The main reason for receiving in-person help was a need for face-to-face contact (47.2%). Many of the IPG and HG participants did not express a need for additional online help when they (had) already received in-person help (72.6%; *N* = 95). However, a majority of survey participants (57.9%; *N* = 107) was still interested in blended care (combining online and in-person support). For the participants who received online help besides in-person help, the main reason was that in-person help did not match their situation or needs (*N* = 5 out of 12).

### Online Help: A Novel Concept With a Broad Definition

Online help was a relatively new and somewhat unfamiliar concept for many participants and they defined a broad range of potential meanings and functions in the context of IPVA. Survey participants mainly used an internet search engine (58.7%) and the VT chat (32.6%). Nine participants mentioned other options, such as 113 Suicide Prevention, Victim Support Netherlands and The Disappeared Self.

Interviewees discussed many variations of online help, such as online support from DVA organizations, fellow survivors, eHealth modules or online interventions, and via social media. Nondigital remote help (via telephone) was discussed as well and some also classified only seeking information and help options online as online help. Many women presented a lack of familiarity with online help.

Online help is still a bit abstract to me. The information provision is very good but to really seek contact …. Because maybe I don’t really know what to expect. Also, sometimes I don’t have a concrete question because I don’t know what's possible. While if I speak with [DVA organization], they mention an e-mail address and then I think oh yeah, that's also a good idea. (p. 108)

In-person help has many variations as well, for example, shelter, therapy, and legal support from various organizations and authorities. Survey and interview participants mentioned similar sources of in-person help and survey participants mostly sought help from a psychologist (*N* = 66 out of 95). While participants were more familiar with these support options, they did stress the need for more information on what to expect from (face-to-face) professional help.

#### Blended Care

**Figure 1.**
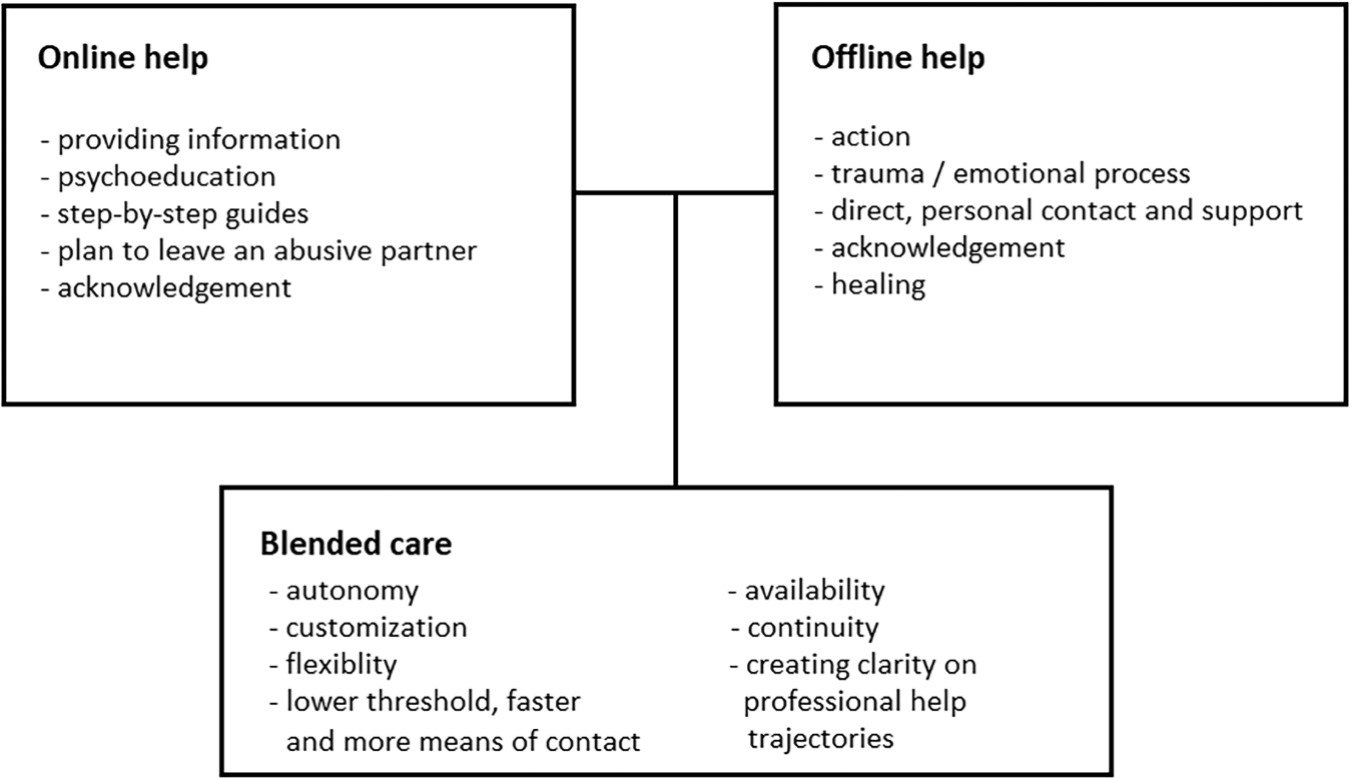
The roles of online and offline help and the added value and potential of blended care.

Almost 60% of survey participants expressed an interest in blended care and, together with the interviewees, presented concrete ideas about how to give substance to it. For example, they found online help was suitable for providing information and step-by-step guides, while in-person help was needed for taking action and when working on an emotional process. Furthermore, blended care could support autonomy and flexibility, and create clarity on professional help trajectories (Figure 1).

(Psycho-)education and step-by-step plans etcetera can often be done quite well online. Most other help requires a listening ear and customized solutions. (p. 213)

For me, online was more to get a confirmation that what happened was not ‘normal’. With in-person help I’ve really grown and I’ve learned a lot. (p. 165)

A few interviewees mentioned having to use online help due to the COVID-19 pandemic and that, as a consequence, blended care was now more normalized. Online tools could function as a stepping stone toward in-person help and provide insight into what to expect from it. While a few interview participants preferred getting to know a professional or fellow survivors in person first, one woman said that in hindsight she preferred the group sessions starting out online because it created a safe distance to share and get to know each other before meeting face-to-face.

Online and in-person help needed to be well-connected and perhaps synchronized according to the interviewees. Furthermore, they stated that continuity in (online) contact with a professional was important. Women discussed their experiences with blended care or explained how they would like it to be arranged, such as using online modules and subsequently discussing it with a professional, or face-to-face sessions with a professional and online contact with fellow survivors (Supplemental File A.3). Interview participants who were not interested in blended care mentioned obstacles similar to those for online and in-person support and felt the combination could be overwhelming or too burdensome.

I think therapy needs to be more personalized and you need help specified to your situation. But if you, for example, have recurring problems with managing stress, I think you can train it through exercises. If you have it on your phone, in an app, and you get daily reminders, I think you’d be much more likely to do it than when a psychologist that you see once per month tells you to do it every day. I think it's a strength if you have both. (p. 101)

## Discussion

This study investigated the interplay between online and in-person help for women IPVA survivors. It also focused on optimizing the use of both options to fit the processes that survivors go through during and in the aftermath of an abusive relationship.

We identified needs, obstacles, and (dis)advantages for help-seeking. In general, shame was a major obstacle and acknowledgment was a prominent need (Rempel et al., 2019; Robinson et al., 2020; Tarzia et al., 2017). Examples of an obstacle and advantage specifically for online help were safety concerns and promoting awareness. Examples of in-person help were practical obstacles and receiving immediate care. In terms of type of contact, it seems online help fits well with a need for anonymity and in-person help with a need for building trust with a professional. Thus, while both types of support address similar needs and obstacles overall, they also harbor specific opportunities and difficulties (Nelson et al., 2022; Van Gelder et al., 2021b).

In-person help was used more than online help, this is mostly due to its relative novelty and participants’ lack of familiarity. Younger women were more likely to use online help and this finding is not unexpected: for some participants, this type of support was not yet available when they sought help and the younger generation is more digitally skilled and used to being online (Centraal Bureau voor de Statistiek, 2021, 2022). Certain types of IPVA seemed to be linked to specific types of help. Significant positive, albeit weak, correlations were found for economic abuse and a DVA organization's online chat; physical violence and the police; sexual violence and a DVA organization; and psychological IPVA and a psychologist. A weak significant negative correlation was found between physical violence and help-seeking via an internet search engine. It could be the case that experiencing physical types of IPVA may lead survivors to seek other types of help than experiencing nonphysical types (Akkermans et al., 2020; Lelaurain et al., 2017).

Since both help types have their own (dis)advantages and some might be more suitable for certain IPVA experiences, a more integrated approach of in-person and online support could improve survivors’ help-seeking and -receiving processes. However, we only found significant correlations within the two help types, not between them. For example, online VT support was linked to a DVA organization's online support, and in-person VT support was linked to a DVA organization's in-person support, police and/or social work. Thus, online and in-person help are not yet optimally complementing each other and integrated into one system. Our study showed fragmented help-seeking journeys as women experienced multiple IPVA types, received multiple forms of support, and stated that the number of professionals involved and the amount of help options can be overwhelming (Lelaurain et al., 2017; Movisie, 2021; Tierolf et al., 2014).

Exploring online help and blended care in the context of IPVA is ongoing. While there was a rapid increase during the COVID-19 pandemic (Brink et al., 2021; Caridade et al., 2021; Van Bemmel et al., 2020; Van Gelder et al., 2021b), many survivors have not yet experienced this type of help in an optimal setting. Nevertheless, participants presented clear ideas on the added value of online help and the interpretation of blended care. For example, online means could play a role in increasing awareness on the various types of IPVA, through providing survivors’ stories and concrete examples (Van Gelder et al., 2021a). Especially for nonphysical (e.g., psychological, economic) IPVA, online platforms could be helpful to validate survivors’ experiences and encourage help-seeking, as for physical and sexual IPVA survivors are more likely to seek in-person help (Akkermans et al., 2020; Lelaurain et al., 2017). Online support could serve as a low-threshold stepping stone in the process of awareness, acknowledgement, and help-seeking (Glass et al., 2021; Gloor & Meier, 2020; Hegarty et al., 2019; Tarzia et al., 2018), especially for people identifying as LGBTIQA+; with a cultural minority background; and/or with a disability (Nelson et al., 2022). With regard to blended care, a majority of the survey participants (57.9%) expressed interest in combining help types because they felt it could increase their autonomy, it is more flexible, and it could prepare survivors for engaging with in-person help (Van Gelder et al., 2021a).

### Limitations

A limitation is the limited demographic data that we gathered to keep the burden for participants to a minimum. For example, we did not collect data on cultural background. Furthermore, this study was conducted online and in Dutch and contained a relatively highly educated sample. The recruitment mainly took place via online means and the data was completely gathered via online means. Although in the Netherlands almost 80% of people between 16 and 75 years old have at least basic digital skills (Centraal Bureau voor de Statistiek, 2022), a consequence of using online means is that women who were not sufficiently digitally literate could not take part in this study. Thus, their views on using online and in-person support are lacking, while this could be of significant influence in their help-seeking process and use of various types of help. Therefore, the outcomes are not fully representative of women IPVA survivors in the Netherlands. Furthermore, no men IPVA survivors were included but their experiences and opinions are important and could differ from women IPVA survivors (Taylor et al., 2022).

Also, no widely accepted, concrete definition has been established yet for online help in the context of IPVA and therefore it can include many forms (El Morr & Layal, 2020; Emezue & Bloom, 2021; Linde et al., 2020). This complicates comparing experiences and essential features of online and blended support, and determining when to provide a particular type of help.

### Implications

Implementing a blended care approach for IPVA survivors who are willing and have the means to use online help can improve the support they receive. Online tools could lower barriers to help-seeking, include harder-to-reach groups, and benefit people who face obstacles to in-person help (Nelson et al., 2022; Tarzia et al., 2017; Van Gelder et al., 2021a). As survivors seem more likely to seek help online (first) for nonphysical abuse and they may have more doubts about whether these “count” as violence, online platforms could be modified to address these types specifically without neglecting physical and sexual IPVA. For emotional processes, it seems that, for at least part of IPVA survivors, this cannot be done solely online, although there are signs of suitable online therapies (Andersson et al., 2021; Tarzia et al., 2018; Van Gelder et al., 2021b). The help-seeking journeys of survivors of IPVA show a lot of variation and complexity and thus both online and in-person types of support need to be tailored to accommodate this (Lelaurain et al., 2017).

We are all exploring online and blended care's possibilities, boundaries, and requirements for an optimal setting. Participants often suggested online help as a means that they can use independently, a way to obtain information and be more flexible, and to meet other survivors. In an optimal setting, the execution of blended care is tailored to the survivor's needs, it is easy and safe to use, and professionals received training on using online tools to aid survivors (Al-Alosi, 2020; El Morr & Layal, 2020; Van Gelder et al., 2021b; Williams et al., 2021). Online tools should be consistently available throughout the process of becoming aware of the abuse till being in the aftermath of having left an abusive relationship (Rempel et al., 2019).

While survivors and professionals are interested in the added value of online and blended care (Covers et al., 2022; Gloor & Meier, 2020; Van Gelder et al., 2021b), this study did highlight a concern: what if it increases the number of options and professionals involved, making it harder for survivors to navigate and increasing the risk of “help fatigue”? With the survivors’ needs for knowing what to expect from (offline) professional help, continuity, and a case manager, online support could be integrated into the system to benefit the survivor instead of adding to the burden. For example, DVA organizations could inform survivors in a low-threshold manner with a flowchart on their website, explaining which support they offer, what it entails, and what happens when a survivor contacts them. Online help could be implemented in the ongoing development of a system-oriented, integrated, multidisciplinary approach to combat IPVA. A case manager managing the involved professionals and authorities could also organize blended care (Lünnemann & Lünnemann, 2022; Regioplan, 2021; Vogtländer & Van Arum, 2016). Furthermore, specialized centers, such as Filomena Domestic Violence and Child Abuse Center and Sexual Assault Center, combining various disciplines in one location could be suitable settings for providing online and blended care (Covers et al., 2022).

Future research should focus on: the definition of online help in the IPVA context; how IPVA survivors navigate various types of online, in-person, and blended care; and assessing the needs, wishes, and obstacles of survivors and professionals to understand the optimal setting for implementing online and blended care. Furthermore, diversity on various levels (e.g., cultural background, sexual orientation, gender identity) is key. As survivors and professionals express a need for online and blended care, and it is clear that these help types could complement each other, it is of great importance to continue the research and development and apply these insights into policy and practice.

## Supplemental Material

sj-docx-1-vaw-10.1177_10778012241270223 - Supplemental material for Navigating Online and in-Person Support: Views and Experiences From Survivors of Intimate Partner Violence and Abuse Supplemental material, sj-docx-1-vaw-10.1177_10778012241270223 for Navigating Online and in-Person Support: Views and Experiences From Survivors of Intimate Partner Violence and Abuse by Nicole van Gelder, Jeyna Sow, Ditte van Haalen, Iris Schoorlemmer, Margreet Knol, Eva Bouwer and Sabine Oertelt-Prigione in Violence Against Women

## Notes

1. To compute correlations for online and in-person help, only participants who received this type of help were selected to limit skewness from responses of participants who did not receive that type of help.

2. To compute correlations with regard to offline help, only participants who ever received offline help were selected. This was done to limit skewness because of responses of participants who never received offline help.

## References

[bibr1-10778012241270223] Akkermans M. Gielen W. Kloosterman R. Moons E. Reep C. Wingen M . (2020). *Prevalentiemonitor Huiselijk Geweld en Seksueel Geweld* 2020. https://www.cbs.nl/nl-nl/publicatie/2020/51/prevalentiemonitor-huiselijk-geweld-en-seksueel-geweld-2020-

[bibr2-10778012241270223] Al-Alosi H. (2020). Fighting fire with fire: Exploring the potential of technology to help victims combat intimate partner violence. Aggression and Violent Behavior, 52, Article 101376. 10.1016/j.avb.2020.101376

[bibr3-10778012241270223] Ali P. A. Dhingra K. McGarry J. (2016). A literature review of intimate partner violence and its classifications. Aggression and Violent Behavior, 31, 16–25. 10.1016/j.avb0.2016.06.008

[bibr4-10778012241270223] Andersson G. Olsson E. Ringsgard E. Sandgren T. Viklund I. Andersson C. Hesselman Y. Johansson R. Nordgren L. B. Bohman B. (2021). Individually tailored internet-delivered cognitive-behavioral therapy for survivors of intimate partner violence: A randomized controlled pilot trial. Internet Interventions, 26, Article 100453. 10.1016/j.invent.2021.100453 34584851 PMC8452796

[bibr5-10778012241270223] Ayres L . (2014). Thematic coding and analysis. The SAGE encyclopedia of qualitative research methods. Sage Publications, Inc. 10.4135/9781412963909

[bibr6-10778012241270223] Black M. C. (2011). Intimate partner violence and adverse health consequences. American Journal of Lifestyle Medicine, 5, 428–439. 10.1177/1559827611410265

[bibr7-10778012241270223] Brink J. Cullen P. Beek K. Peters S. A. E. (2021). Intimate partner violence during the COVID-19 pandemic in Western and Southern European countries. European Journal of Public Health, 31, 1058–1063. 10.1093/eurpub/ckab093 34406373 PMC8436372

[bibr8-10778012241270223] Caridade S. M. M. Saavedra R. Ribeiro R. Oliveira A. C. Santos M. Almeida I. S. Soeiro C. (2021). Remote support to victims of violence against women and domestic violence during the COVID-19 pandemic. The Journal of Adult Protection, 23, 302–316. 10.1108/jap-04-2021-0015

[bibr9-10778012241270223] Centraal Bureau voor de Statistiek. (2021). *Internettoegang en internetactiviteiten; persoonskenmerken* . https://www.cbs.nl/nl-nl/cijfers/detail/84888NED

[bibr10-10778012241270223] Centraal Bureau voor de Statistiek. (2022). *Nederland Europese koploper digitale vaardigheden*. https://www.cbs.nl/nl-nl/nieuws/2022/19/nederland-europese-koploper-digitale-vaardigheden

[bibr11-10778012241270223] Covers M. Noteboom F. Dam A. Bicanic A. (2022). *Drempels voor disclosure van seksueel geweld in anonieme online hulpverlening*. https://centrumseksueelgeweld.nl/wp-content/uploads/2022/06/CSG_Rapport_Drempels-voor-disclosure.pdf

[bibr12-10778012241270223] Ellsberg M. Jansen H. A. Heise L. Watts C. H. Garcia-Moreno C. (2008). Intimate partner violence and women's physical and mental health in the WHO multi-country study on women's health and domestic violence: An observational study. The Lancet, 371, 1165–1172. 10.1016/S0140-6736(08)60522-X 18395577

[bibr13-10778012241270223] El Morr C. & Layal M. (2020). Effectiveness of ICT-based intimate partner violence interventions: A systematic review. BMC Public Health, 20, Article 1372. 10.1186/s12889-020-09408-8 32894115 PMC7476255

[bibr14-10778012241270223] Emezue C. (2020). Digital or digitally delivered responses to domestic and intimate partner violence during COVID-19. JMIR public Health and Surveillance, 6, Article e19831. 10.2196/19831 PMC7394520 32678797

[bibr15-10778012241270223] Emezue C. Bloom T. L. (2021). Protocol: Technology-based and digital interventions for intimate partner violence: A meta-analysis and systematic review. Campbell Systematic Reviews, 17, Article e1132. 10.1002/cl2.1132 PMC8356356 37050972

[bibr16-10778012241270223] Evans M. A. Feder G. S. (2016). Help-seeking amongst women survivors of domestic violence: A qualitative study of pathways towards formal and informal support. Health Expectations, 19, 62–73. 10.1111/hex.12330 25556776 PMC5055220

[bibr17-10778012241270223] Ford-Gilboe M. Varcoe C. Scott-Storey K. Perrin N. Wuest J. Wathen C. N. Case J. Glass N. (2020). Longitudinal impacts of an online safety and health intervention for women experiencing intimate partner violence: Randomized controlled trial. BMC Public Health, 20, Article 260. 10.1186/s12889-020-8152-8 32098633 PMC7043036

[bibr18-10778012241270223] Friese S. (2011). Qualitative data analysis with ATLAS.ti. Sage Publications.

[bibr19-10778012241270223] Garnweidner-Holme L. Henriksen L. Flaathen E. M. Klette Bohler T. Lukasse M. (2020). Midwives’ attitudes toward and experience with a tablet intervention to promote safety behaviors for pregnant women reporting intimate partner violence: Qualitative study. JMIR mHealth and UHealth, 8, Article e16828. 10.2196/16828 PMC7270855 32432553

[bibr20-10778012241270223] Glass N. E. Clough A. Messing J. T. Bloom T. Brown M. L. Eden K. B. Campbell J. C. Gielen A. Laughon K. Grace K. T. Turner R. M. Alvarez C. Case J. Barnes-Hoyt J. Alhusen J. Hanson G. C. Perrin N. A. (2021). Longitudinal impact of the myPlan app on health and safety among college women experiencing partner violence. Journal of Interpersonal Violence, 37, NP11436–NP11459. 10.1177/0886260521991880 33576291

[bibr21-10778012241270223] Gloor D. Meier H. (2020). Does online counselling provide better access to victim services? Insights and reflections from a Swiss pilot evaluation. Journal of Gender-Based Violence, 4, 123–131. 10.1332/239868020×15794803790621

[bibr22-10778012241270223] Hegarty K. Tarzia L. Valpied J. Murray E. Humphreys C. Taft A. Novy K. Gold L. Glass N. (2019). An online healthy relationship tool and safety decision aid for women experiencing intimate partner violence (I-DECIDE): A randomised controlled trial. The Lancet Public Health, 4, e301–e310. 10.1016/s2468-2667(19)30079-9 31155223

[bibr23-10778012241270223] Henning E. Van Rensburg W. Smit B. (2004). Making meaning of data: Analysis and interpretation. In Finding your way in qualitative research (pp. 354–504). Van Schaik Publishers.

[bibr24-10778012241270223] Hennink M. M. Kaiser B. N. Marconi V. C. (2017). Code saturation versus meaning saturation: How many interviews are enough? Qualitative Health Research, 27, 591–608. 10.1177/1049732316665344 27670770 PMC9359070

[bibr25-10778012241270223] IBM Corp. (2017). IBM SPSS statistics for windows. (Version 25.0) [Computer software].

[bibr26-10778012241270223] Johnson L. Chen Y. Stylianou A. Arnold A. (2022). Examining the impact of economic abuse on survivors of intimate partner violence: A scoping review. BMC Public Health, 22, Article 1014. 10.1186/s12889-022-13297-4 35590302 PMC9121607

[bibr27-10778012241270223] Lelaurain S. Graziani P. Lo Monaco G. (2017). Intimate partner violence and help-seeking. European Psychologist, 22, 263–281. 10.1027/1016-9040/a000304

[bibr28-10778012241270223] Linde D. S. Bakiewicz A. Normann A. K. Hansen N. B. Lundh A. Rasch V. (2020). Intimate partner violence and electronic health interventions: Systematic review and meta-analysis of randomized trials. Journal of Medical Internet Research, 22, Article e22361. 10.2196/22361 PMC7762681 33306030

[bibr29-10778012241270223] Lünnemann K. Lünnemann M. (2022). *Integrale behandeling van gezinsleden bij huiselijk geweld: procesevaluatie Switch*. https://www.verwey-jonker.nl/publicatie/integrale-behandeling-van-gezinsleden-bij-huiselijk-geweld/

[bibr30-10778012241270223] Movisie (2021). *Meer concrete kennis nodig over huiselijk geweld en kindermishandeling*. https://www.movisie.nl/artikel/meer-concrete-kennis-nodig-over-huiselijk-geweld-kindermishandeling

[bibr31-10778012241270223] Nelson A. Allen J. Cho H. Yun S. H. Choi Y. J. Choi G.-Y. (2022). Intimate partner violence and openness to online counseling among college students. Journal of Family Violence, 38, 611–621. 10.1007/s10896-022-00396-4 35464668 PMC9016205

[bibr32-10778012241270223] O’Doherty L. J. Taft A. McNair R. Hegarty K. (2016). Fractured identity in the context of intimate partner violence: Barriers to and opportunities for seeking help in health settings. Violence Against Women, 22, 225–248. 10.1177/1077801215601248 26337674

[bibr33-10778012241270223] Regioplan. (2021). ‘*Niet samenwerken omdat het moet, maar omdat het helpt’ - Domeinoverstijgend werken in het lokaal veld rondom huiselijk geweld en kindermishandeling – leren van Rotterdam-Rijnmond en Gooi en Vechtstreek*. https://www.huiselijkgeweld.nl/dossiers/programma-geweld-hoort-nergens-thuis/publicaties/rapporten/2021/12/08/niet-samenwerken-omdat-het-moet-maar-omdat-het-helpt

[bibr34-10778012241270223] Rempel E. Donelle L. Hall J. Rodger S. (2019). Intimate partner violence: A review of online interventions. Informatics for Health and Social Care, 44, 204–219. 10.1080/17538157.2018.1433675 29537928

[bibr35-10778012241270223] Robinson S. R. Ravi K. Voth Schrag R. J. (2020). A systematic review of barriers to formal help seeking for adult survivors of IPV in the United States, 2005-2019. Trauma, Violence & Abuse, 22, 1279–1295. https://doi.org/1524838020916254 10.1177/1524838020916254 32266870

[bibr36-10778012241270223] Römkens R. de Jong T. Harthoorn H . (2016). *Violence against women Dutch context - European Union survey results in the Dutch context*. https://collectie.atria.nl/bibliotheek/item/210786-violence-against-women

[bibr37-10778012241270223] Tarzia L. Cornelio R. Forsdike K. Hegarty K. (2018). Women’s experiences receiving support online for intimate partner violence: How does it compare to face-to-face support from a health professional? Interacting with Computers, 30, 433–443. 10.1093/iwc/iwy019

[bibr38-10778012241270223] Tarzia L. Iyer D. Thrower E. Hegarty K. (2017). ‘Technology doesn’t judge you’: Young Australian women’s views on using the internet and smartphones to address intimate partner violence. Journal of Technology in Human Services, 35, 199–218. 10.1080/15228835.2017.1350616

[bibr39-10778012241270223] Taylor J. C. Bates E. A. Colosi A. Creer A. J. (2022). Barriers to men's help seeking for intimate partner violence. Journal of Interpersonal Violence, 37, NP18417–NP18444. 10.1177/08862605211035870 PMC9554285 34431376

[bibr40-10778012241270223] Ten Boom A. Wittebrood K. (2019). *De prevalentie huiselijk geweld en kindermishandeling in Nederland*. https://www.huiselijkgeweld.nl/publicaties/rapporten/2019/02/06/de-prevalentie-van-huiselijk-geweld-en-kindermishandeling-in-nederland

[bibr41-10778012241270223] Tierolf B. Lünnemann K. Steketee M. (2014). *Doorbreken geweldspatroon vraagt gespecialiseerde hulp: Onderzoek naar effectiviteit van de aanpak huiselijk geweld in de G4*. https://www.verwey-jonker.nl/publicatie/doorbreken-geweldspatroon-vraagt-gespecialiseerde-hulp/

[bibr42-10778012241270223] Van Bemmel S. R. Simons E. I. Noteboom F. (2020). *Effecten van corona: Een analyse op basis van de digitale hulpverlening verzorgd door ‘Chat met Fier’*. https://www.huiselijkgeweld.nl/publicaties/rapporten/2020/09/24/chat-met-fier-en-de-effecten-van-corona

[bibr43-10778012241270223] Van Eijkern L. Downes R. Veenstra R. (2018). *Slachtofferschap van huiselijk geweld: Prevalentieonderzoek naar de omvang, aard, relaties en gevolgen van slachtoffer- en plegerschap*. https://repository.wodc.nl/handle/20.500.12832/2239

[bibr44-10778012241270223] Van Gelder N. Ligthart S. Ten Elzen J. Prins J. van Rosmalen-Nooijens K. Oertelt-Prigione S. (2021a). If i'd had something like SAFE at the time, maybe I would've left him sooner.- essential features of eHealth interventions for women exposed to intimate partner violence: A qualitative study. Journal of Interpersonal Violence, 37, NP18341–NP18375. 10.1177/08862605211036108 PMC9554282 34355982

[bibr45-10778012241270223] Van Gelder N. E. van Haalen D. L. Ekker K. Ligthart S. A. Oertelt-Prigione S. (2021b). Professionals’ views on working in the field of domestic violence and abuse during the first wave of COVID-19: A qualitative study in the Netherlands. BMC Health Services Research, 21, Article 624. 10.1186/s12913-021-06674-z 34193134 PMC8241882

[bibr46-10778012241270223] Van Gelder N. E. van Rosmalen-Nooijens K. A. W. L. Ligthart A. Prins S. Oertelt-Prigione J. B. & Lagro-Janssen S. M A. L. (2020). SAFE: An eHealth intervention for women experiencing intimate partner violence – study protocol for a randomized controlled trial, process evaluation and open feasibility study. BMC Public Health, 20, Article 640. 10.1186/s12889-020-08743-0 32380972 PMC7204286

[bibr47-10778012241270223] Vogtländer L. van Arum S. (2016). *Eerst samenwerken voor veiligheid, dan samenwerken voor risicogestuurde zorg*. https://vng.nl/files/vng/201605_visiedocument_gefaseerde_ketensamenwerkingvogtlander_van_arum_0.pdf

[bibr48-10778012241270223] Williams E. E. Arant K. R. Leifer V. P. Balcom M. C. Levy-Carrick N. C. Lewis-O’Connor A. Katz J. N. (2021). Provider perspectives on the provision of safe, equitable, trauma-informed care for intimate partner violence survivors during the COVID-19 pandemic: A qualitative study. BMC Women's Health, 21, Article 315. 10.1186/s12905-021-01460-9 34452616 PMC8393774

[bibr49-10778012241270223] World Health Organization. (2021). *Violence against women prevalence estimates 2018*. https://www.who.int/publications/i/item/9789240022256

