# Supplementary material for: Navigating Online and in-Person Support: Views and Experiences From Survivors of Intimate Partner Violence and Abuse
Source: Violence Against Women. 2024 Aug 8;31(11):2957–75. doi: 10.1177/10778012241270223 (PMC12241680; doi:10.1177/10778012241270223)
Supplement: sj-docx-1-vaw-10.1177_10778012241270223 - Supplemental material for Navigating Online and in-Person Support: Views and Experiences From Survivors of Intimate Partner Violence and Abuse [file sj-docx-1-vaw-10.1177_10778012241270223.docx]

**Table A.1.** Survey participants (N=107) demographics.

| **Age** | **Educational level** | **Province** | **IPVA type** | **Type of support** |
| --- | --- | --- | --- | --- |
| ***Mean age =*** 43 | ***Secondary education***: N=4 | ***Drenthe:*** N=2 | ***Psychological:*** N=105 | ***Offline only:*** N=53 |
| ***Range =*** 21 – 74 | ***Vocational education:*** N=37 | ***Flevoland:*** N=5 | ***Physical:*** N=82 | ***Online only:*** N=4 |
| ***Age category*** | ***Higher vocational education:*** N=49 | ***Friesland:*** N=1 | ***Economical:*** N=61 | ***Hybrid (online and offline):*** N=42 |
| ***21 – 29:*** N=11 | ***University:*** N=16 | ***Gelderland:*** N=14 | ***Sexual:*** N=44 | ***No help:*** N=8 |
| ***30 – 39:*** N=27 | ***Postdoctoral:*** N=1 | ***Groningen:*** N=5 |  |  |
| ***40 – 49:*** N=40 |  | ***Limburg:*** N=3 |  |  |
| ***50 – 59:*** N=25 |  | ***Noord-Brabant:*** N=11 |  |  |
| ***60 – 69:*** N=3 |  | ***Noord-Holland:*** N=14 |  |  |
| ***70 – 74:*** N=1 |  | ***Overijssel:*** N=16 |  |  |
|  |  | ***Utrecht:*** N=6 |  |  |
|  |  | ***Zeeland:*** N=5 |  |  |
|  |  | ***Zuid-Holland:*** N=25 |  |  |

**Table A.2.** Interview participants (N=18) demographics.

| **Age** | **Educational level** | **Province** | **IPVA type** | **Type of support** |
| --- | --- | --- | --- | --- |
| ***Mean age:*** 43 | ***Secondary education***: N=1 | ***Drenthe:*** N=0 | ***Psychological:*** N=18 | ***Offline only:*** N=2 |
| ***Range:*** 26 – 75 | ***Vocational education:*** N=6 | ***Flevoland:*** N=1 | ***Physical:*** N=16 | ***Online only:*** N=0 |
| ***Age category*** | ***Higher vocational education:*** N=11 | ***Friesland:*** N=0 | ***Economical:*** N=10 | ***Hybrid (online and offline):*** N=16 |
| ***26 – 29:*** N=2 | ***University:*** N=0 | ***Gelderland:*** N=4 | ***Sexual:*** N=11 | ***No help:*** N=0 |
| ***30 – 39:*** N=5 | ***Postdoctoral:*** N=0 | ***Groningen:*** N=1 |  |  |
| ***40 – 49:*** N=7 |  | ***Limburg:*** N=0 |  |  |
| ***50 – 59:*** N=3 |  | ***Noord-Brabant:*** N=0 |  |  |
| ***60 – 69:*** N=0 |  | ***Noord-Holland:*** N=3 |  |  |
| ***70 – 75:*** N=1 |  | ***Overijssel:*** N=5 |  |  |
|  |  | ***Utrecht:*** N=0 |  |  |
|  |  | ***Zeeland:*** N=0 |  |  |
|  |  | ***Zuid-Holland:*** N=4 |  |  |

**Table A.3.** Help option correlations in the hybrid help user group (N=42).

|  | **1.** | **2.** | **3.** | **4.** | **5.** | **6.** | **7.** | **8.** | **9.** | **10.** | **11.** |
| --- | --- | --- | --- | --- | --- | --- | --- | --- | --- | --- | --- |
| **1. Internet search engine (Online)** | – |  |  |  |  |  |  |  |  |  |  |
| **2. Chat VT (Online)** | -.372* |  |  |  |  |  |  |  |  |  |  |
| **3. Chat DVA organization (Online)** | -.312* | .433** |  |  |  |  |  |  |  |  |  |
| **4. SAFE (Online)** | .021 | .226 | .000 |  |  |  |  |  |  |  |  |
| **5. Courses or modules (Online)** | -.046 | -.086 | .149 | .271 |  |  |  |  |  |  |  |
| **6. VT (Offline)** | -.272 | .069 | .100 | .175 | -.006 |  |  |  |  |  |  |
| **7. DVA organization (Offline)** | .059 | -.040 | .251 | .200 | .014 | .367* |  |  |  |  |  |
| **8. GP (family doctor) or GP office’s mental health worker (Dutch: *POH-GGZ*) (Offline)** | .225 | -.240 | -.079 | -.022 | .276 | .048 | -.108 |  |  |  |  |
| **9. Police (Offline)** | -.091 | .034 | -.117 | -.043 | -.098 | .528** | .139 | .106 |  |  |  |
| **10. Social work / Social team (Dutch: *buurtteam / sociaal team*) / Centre for Children and Families (Dutch: *Centrum voor Jeugd en Gezin*) (Offline)** | -.100 | -.034 | -.019 | .171 | .220 | .355* | .085 | .379* | .336* |  |  |
| **11. Psychologist (Offline)** | -.165 | .171 | .198 | .108 | .029 | .147 | .005 | .209 | .203 | .379* |  |
| **12. Sexual Assault Centre (Dutch: *Centrum Seksueel Geweld*) (Offline)** | .142 | -.110 | -.064 | -.070 | -.076 | .199 | .279 | .129 | .164 | -.164 | .129 |

| *Note:* interactions between offline and online help types were analyzed in one model with Pearson’s correlations. Only HG (N=42) was included as all  participants in this group used both online and offline help. \| *Correlation is significant at the 0.05 level (2-tailed). \| **Correlation is significant at the  0.01 level (2-tailed). |
| --- |

**Figure A.1.** The roles of online and offline help and the added value and potential of blended care.
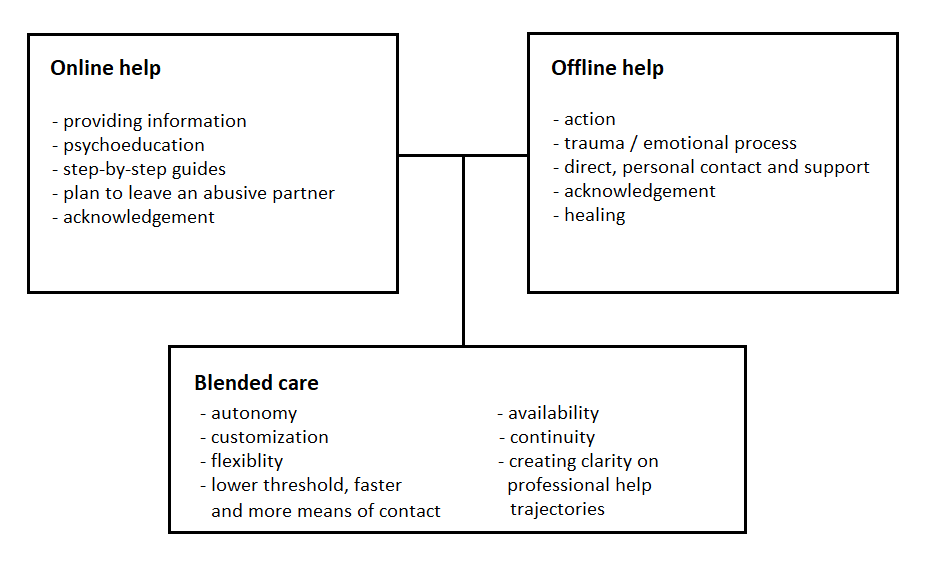


**Supplementary file A.1 – Translated online survey.**

(We only included the content related questions in this translation.)

1. (All groups) Have you ever used online help for IPVA? For example, an online chat (such as with *Fier* or *Veilig Thuis*) or a website / online platform such as www.safewomen.nl. [Multiple answers possible]
   1. No, I have never used online help and have never sought help online.

🡪 Question 2 (all yes answers go to question 3)

- 1. Yes, I have used Google (or another search engine) to seek help.
  2. Yes, I have used the chat from Veilig Thuis.
  3. Yes, I have used the chat of DVA organization such as Fier of Moviera.
  4. Yes, I have used www.safewomen.nl.
  5. Yes, I have used online help in the form of a course or module(s). This was part of therapy or the help trajectory that I received because of IPVA.
  6. Yes. I have used another online option, namely: [type answer].

1. (Offline only) Why have you never used online help and never searched online for help? [Multiple answers possible]
2. I found help via other means, for example, the GP, police, Veilig Thuis, a DVA organization, or because 112 was called by myself or someone else.
3. I do / did not know what to look for exactly.
4. I find / found it difficult to use the internet.
5. I do / did not believe I could find something online that could help me.
6. A different reason, namely: [type answer].
7. Another different reason, namely: [type answer].
8. (Online only and hybrid) What was the most important reason for you to seek online help? [Maximum of 2 answers possible]
9. Protecting myself (and your children, other family members, or pets).
10. A crisis situation, for example (severe) physical assault or threats.
11. To connect with other survivors of IPVA.
12. Acknowledging that you could not solve the situation by yourself.
13. To seek information on which support options are available.
14. To seek information on IPVA because you had doubts about if what you were / are experiencing is IPVA.
15. Other, namely: [type answer].
16. (Online only and hybrid) What do / did you need most when using online support? [Maximum of 2 answers possible]
17. Someone who listens, acknowledgement.
18. Concrete tips.
19. Direct (acute) care or help.
20. Contact with other people in the same situation.
21. Other, namely: [type answer].
22. (Online only and hybrid) How did you find this type of help?
23. By searching online yourself, for example via Google.
24. Via your own social network, for example a family member, friend or colleague.
25. Via your GP or mental health practitioner.
26. Via the police.
27. Via your psychologist.
28. Via a social worker / social team / Centre for Children and Families.
29. Via Veilig Thuis.
30. Via a DVA organization, for example Kadera, BlijfGroep, Fier.
31. Other, namely: [type answer].
32. (Online only and hybrid) How difficult was it for you to seek / start using online help?
33. Very difficult
34. Difficult
35. Neutral
36. Easy
37. Very easy
38. (Online only and hybrid) The online support met / meets my expectations:
39. Totally agree
40. Agree
41. Neutral
42. Disagree
43. Totally disagree
44. (All groups) Did / do you experience obstacles to seek online help? [Multiple answers possible]
45. No, I have not experienced obstacles in seeking online help or seeking help via the internet.
46. Yes, because I feel / felt it is impersonal.
47. Yes, because I am / was worries about my privacy and anonymity.
48. Yes, because I am / was worried about my safety. For example, I am / was worried someone finds out I am searching for online help.
49. Yes, because I am / was not familiar with online help and I do / did not know what to expect from it.
50. Yes, because I feel / felt that my problems are not serious / severe enough.
51. Yes, because I feel / felt ashamed about what I experienced.
52. Yes, because I feel / felt the violence or abuse by my (ex-)partner is / was my own fault.
53. (Online only and hybrid) Did / do you also use offline (in-person) help next to online help?
    1. No, I do / did not need it.

🡪 All no answers go to question 11.

- 1. No, not yet but I am thinking about it.
  2. Yes, via online help I went to a DVA organization, for example Kadera, BlijfGroep, Fier.

🡪 All yes answers go to question 10.

- 1. Yes, via online help I went to Veilig Thuis.
  2. Yes, via online help I went to the GP, mental health practitioner or psychologist.
  3. Yes, via online help I went to social work / social team / Centre for Children and Families.

1. (Hybrid) Why do / did you also use offline support? [Multiple answers possible]
2. I find / found that offline help is a good addition to online help.
3. The information I receive(d) via online help is / was not sufficient.
4. I do / did not like using online help, I rather use offline help.
5. I need(ed) personal (in-person) contact with someone.
6. The online help I receive(d) did / does not match my situation / need for help.
7. My situation has changed, causing the online help to not match my need for help anymore.
8. Other, namely: [type answer].
9. (All groups) According to you, what could be the advantages and disadvantages of online help? [open question]

(All groups) The next questions are about experiences with offline (in-person) help. If you do not have experience with this type of help we still have a few questions we want to ask.

1. (All groups) Have you ever used offline (in-person) help for IPVA? For example, shelter, in-person therapy via a DVA organization. [Multiple answers possible]
2. No, I have never used offline help.
3. Yes, I have used help from Veilig Thuis.
4. Yes, I have used help from a DVA organization, for example Kadera, BlijfGroep, Fier.
5. Yes, I have used help from my GP or a mental health practitioner.
6. Yes, I have used help from the police.
7. Yes, I have used help from s social worker / social team / Centre for Children and Families.
8. Yes, I have used help from a psychologist.
9. Yes, I have used help from the Sexual Assault Center.
10. Yes, I have used another type of offline help, namely: [type answer].
11. (Offline only and hybrid) What was the most important reason for you to seek offline help? [Maximum of 2 answers possible]
12. Protecting myself (and your children, other family members, or pets).
13. A crisis situation, for example (severe) physical assault or threats.
14. To connect with other survivors of IPVA.
15. Acknowledging that you could not solve the situation by yourself.
16. To seek information on which support options are available.
17. To seek information on IPVA because you had doubts about if what you were / are experiencing is IPVA.
18. Other, namely: [type answer].
19. (Offline help and hybrid) What do / did you need most when using offline support? [Maximum of 2 answers possible]
20. Someone who listens, acknowledgement.
21. Concrete tips.
22. Direct (acute) care or help.
23. Contact with other people in the same situation.
24. Other, namely: [type answer].
25. (Offline only and hybrid) How did you find this type of help?
26. By searching online yourself, for example via Google.
27. Via your own social network, for example a family member, friend or colleague.
28. Via your GP or mental health practitioner.
29. Via the police.
30. Via a type of online help: the chat from Veilig Thuis.
31. Via a type of online help: the chat from a DVA organization, for example Fier or Moviera.
32. Via the online platform www.safewomen.nl.
33. Via your psychologist.
34. Other, namely: [type answer].
35. (Offline only and hybrid) How difficult was it for you to seek / start using offline help?
36. Very difficult
37. Difficult
38. Neutral
39. Easy
40. Very easy
41. (Offline only and hybrid) The offline support met / meets my expectations:
42. Totally agree
43. Agree
44. Neutral
45. Disagree
46. Totally disagree
47. (All groups) Did / do you experience obstacles to seek offline help? [Multiple answers possible]
48. No, I have not experienced obstacles in seeking offline help.
49. Yes, I experienced practical obstacles, for example a lack of time or suitable help nearby.
50. Yes, because I am / was worries about my privacy and anonymity.
51. Yes, my partner prevents / prevented my from seeking offline help.
52. Yes, because I am / was afraid of the consequences of seeking help, for example having to end my relationship.
53. Yes, because I feel / felt that my problems are not serious / severe enough.
54. Yes, because I feel / felt ashamed about what I experienced.
55. Yes, because I feel / felt the violence or abuse by my (ex-)partner is / was my own fault.
56. (Offline only and hybrid) Did / do you also use online help next to offline help?
    1. No, I do / did not need it. 🡪 No answers go to question 21.
    2. No, not yet but I am thinking about it.
    3. Yes, via online help I went to www.safewomen.nl. 🡪 Yes answers go to question 20.
    4. Yes, via online help I went to an online chat from a DVA organization, for example Fier or Moviera.
    5. Yes, via online help I went to the online chat from Veilig Thuis.
57. (Hybrid) Why do / did you also use online support? [Multiple answers possible]
58. I find / found that online help is a good addition to offline help.
59. Online help is more low-threshold for me.
60. I do / did not like using offline help, I rather use online help.
61. The offline help I receive(d) did / does not match my situation / need for help.
62. My situation has changed, causing the offline help to not match my need for help anymore.
63. Other, namely: [type answer].
64. (All groups) According to you, what could be the advantages and disadvantages of offline help? [open question]
65. (All groups) Would you like to receive help partially online and partially offline (blended)? And could you explain why (not)?
66. Yes, because: [type answer].
67. No, because: [type answer].

**Supplementary file A.2 – Translated semi-structured interview guide.**

(We only included the content related questions in this translation.)

1. (All groups) Have you ever used online help?
2. Answering no 🡪 offline only group:
3. What do you know about online help?
4. Have you ever thought about using online help and why (not)?
5. According to you, what could be the added value of online help on top of in-person (offline) help?
6. Would you like to know more about online help?
7. Answering yes 🡪 online only and hybrid group:
   1. Which type(s) of online help for IPVA have you used?
   2. What were the most important reasons for you to seek online help?
   3. Why did you choose this specific type of support?
8. (Online only and hybrid) Can you tell something about the steps you have taken to get to this online help?
9. When did you start seeking online help? (During or after the abusive relationship?)
10. Did you talk to anyone, for example a family member, friend or colleague, about your situation before seeking online support?
11. (Online only and hybrid) What did you find most difficult about seeking online help?
12. Were there specific obstacles in seeking online help?
13. According to you, what could be done to make seeking online help easier?
14. (Online only and hybrid) What are / were your needs in using online help?
15. What were your expectations of online help? What did you wanted to achieve with it?
16. Did the online help meet your expectations? What did you get out of it?
17. According to you, what is positive about online support?
18. According to you, what is negative about online support?
    1. Do you miss anything in online help?
19. What do you think about the safety and trustworthiness of online help?
20. What do you think about the voice / control that you have / had in using online help?
21. (Online only and hybrid) Have you also used in-person (offline) help? / Do you also use in-person (offline) help?
22. Answering no 🡪 online only group:
23. Have you ever thought about using offline help and why (not)?
24. Has the online help provided you with sufficient insight into the options for offline help?
25. Are there specific barriers in seeking offline help?
26. Answering yes 🡪 hybrid group:
27. When did you start seeking offline help? (Before / during / after using online help?)
28. Why did you choose to use offline help as well?
29. (Offline only and hybrid) Which type(s) of offline help for IPVA have you used?
30. Which organization(s)?
31. What were the most important reasons for you to seek offline help?
32. Why did you choose this specific type of support?
33. (Offline only and hybrid) Can you tell something about the steps you have taken to get to this offline help?
34. When did you start seeking offline help? (During or after the abusive relationship?)
35. Did you talk to anyone, for example a family member, friend or colleague, about your situation before seeking offline support?
36. (Hybrid) Have you used online and offline help simultaneously?
37. What do you think about the connection between offline and online help?
38. Did online help support you in finding offline help and why (not)?
39. Are there things that you miss(ed) in online help that are present in offline help and vice versa?
40. (Offline only and hybrid) What did you find most difficult about seeking offline help?
41. Were there specific obstacles in seeking offline help?
42. According to you, what could be done to make seeking offline help easier?
43. (Offline only and hybrid) What are / were your needs in using offline help?
44. What were your expectations of offline help? What did you wanted to achieve with it?
45. Did the offline help meet your expectations? What did you get out of it?
46. According to you, what is positive about offline support?
47. According to you, what is negative about offline support?
48. Do you miss anything in offline help?
49. What do you think about the safety and trustworthiness of offline help?
50. What do you think about the voice / control that you have / had in using offline help?
51. (All groups) Would you be interested in receiving help partially online and partially offline (blended)?
52. According to you, what could be the added value of the combination of online and offline help?
53. At what time would you feel a need for online help?
54. At what time would you feel a need for offline help?
55. How would blended help look like according to you?
56. What kind of information or help would you like to receive online?
57. What kind of information or help would you like to receive offline?

**Supplementary file A.3. – Appendices.**

**Appendix A.1.** Obstacles and needs of online and offline help.

|  | Online help | Offline help |
| --- | --- | --- |
| Obstacles | - Safety concerns - Lack of personal (‘real’) contact - Harder to share feelings and open up - Not knowing who you talk to / who reads your messages / what happens with the information you share - Traceability - Lack of digital skills - Not knowing online help exists / what to expect from online help | - Practical: lack of time or suitable help nearby - Fear of losing children - Professionals’ lack of expertise / not knowing how to help - Having to leave everything behind when entering a shelter - COVID-19 pandemic |
| Needs | - Receiving specific tips - ‘Real’ interaction - Contact with fellow survivors - Safety - Anonymity - Low-threshold | - Receiving immediate (acute) care / help - Social interaction - Safety - Being taken seriously - Empathy - Having a connection with the professional |

*Note:* additional factors that either positively or negatively influenced their help seeking process, specifically for offline help: their children, partner, finances, pets, cultural or religious background, social life, and the COVID-19 pandemic.

**Appendix A.2.** Advantages and disadvantages of online and offline help.

|  | Online help | Offline help |
| --- | --- | --- |
| Advantages | - Anonymity - Accessible / low-threshold - Fast - Safe - At your own pace - Informative | - Personal - A listening ear - Immediate action - Possibility to build trust - More support |
| Disadvantages | - Traceable - Impersonal - Not safe (privacy) - Hard to use for people without internet or a digital device - Hard to use with a controlling partner - Less connection - More difficult to build trust | - Less accessible / not low-threshold - Logistics - Financial obstacles - Waiting lists - Not anonymous |

**Appendix A.3.** Types of online and offline help used by the survey group (N=99).

|  | **Online help (total N=46)** | **Offline help (total N=95)** |
| --- | --- | --- |
| **Google / another search engine** | N=27 | N/A |
| **VT (*Veilig Thuis*; Safe Home)** | N=16 | N=39 |
| **DVA organization** | N=8 | N=36 |
| **SAFE** | N=8 | N/A |
| **Online courses or modules** | N=9 | N/A |
| **GP or mental health nurse (POH-GGZ)** | N/A | N=56 |
| **Police** | N=1 | N=51 |
| **Social work / social team / Centre for Children and Families** | N/A | N=46 |
| **Psychologist** | N/A | N=66 |
| **Sexual Assault Centre** | N=0 | N=3 |
| **Other*** | N=6 | N=12 |

*Note:* N/A = not applicable, some help types were only available online or offline. | *Other: online = 113 Suicide prevention, Hear My Voice, *Het Verdwenen Zelf*, *Slachtofferhulp Nederland*, Lady’s Linked, Stichting Zijweg; offline = coaches, legal help, psychiatric and trauma help, courses, Lady’s Linked, spiritual guidance, informal support, *Slachtofferhulp Nederland*.

**Appendix A.4.** Examples of blended care from the interview group (N=18).

| - Completing online surveys or modules and subsequently discussing it face-to-face with a psychologist, mental health worker at the GP office, or in a group session with fellow survivors. - Face-to-face counselling once or twice a month and online help the rest of the time. - Discussing things with a counsellor via Whatsapp and following offline courses at a DVA organization. - Face-to-face sessions with a psychologists and contact with fellow survivors via Facebook groups. - Sessions with a therapist could weekly alternate between online / remote and offline. |
| --- |
